# Machine learning-based radiomics modeling of combined subcortical nuclei from quantitative susceptibility mapping for Parkinson's disease diagnosis

**DOI:** 10.3389/fnagi.2026.1869390

**Published:** 2026-08-11

**Authors:** Fudong Wen, Jiawei Hu, Yubing Yang, Yue Su, Xinyu Song, Yuan Tian, Yupeng Wang

**Affiliations:** 1 Department of Biostatistics, Public Health College, Harbin Medical University, Harbin, Heilongjiang, China; 2 Department of Epidemiology, Public Health College, Harbin Medical University, Harbin, Heilongjiang, China; 3 The First Affiliated Hospital of Harbin Medical University, Harbin, Heilongjiang, China; 4 Department of Magnetic Resonance, The First Affiliated Hospital of Harbin Medical University, Harbin, Heilongjiang, China

**Keywords:** machine learning, Parkinson's disease, quantitative susceptibility mapping, radiomics, subcortical nuclei

## Abstract

**Background:**

Quantitative susceptibility mapping (QSM) enables non-invasive assessment of brain iron deposition in Parkinson's disease (PD), yet existing radiomics studies have predominantly focused on single subcortical nuclei, without systematically evaluating whether combining multiple regions improves diagnostic accuracy.

**Methods:**

A total of 59 PD patients and 73 healthy controls underwent QSM. Radiomic features were extracted from the substantia nigra (SN), caudate nucleus (CN), globus pallidus (GP), red nucleus (RN), and putamen (Put). For all 31 possible combinations of the five nuclei, feature selection was performed on the training set using Wilcoxon rank-sum tests, minimal redundancy maximal relevance, and least absolute shrinkage and selection operator regression. Four classifiers—logistic regression (LR), support vector machine (SVM), random forest (RF), and extreme gradient boosting (XGBoost)—were trained and evaluated under a stratified five-fold nested cross-validation framework. Diagnostic performance was assessed primarily using the area under the receiver operating characteristic curve (AUC).

**Results:**

LR achieved its best mean test AUC of 0.879 ± 0.045 with CN + GP. SVM and RF yielded optimal mean test AUCs of 0.880 ± 0.047 (GP + RN + Put) and 0.891 ± 0.028 (CN + GP), respectively. XGBoost with GP + RN + Put produced the highest mean test AUC among all models (0.921 ± 0.040) and was designated the global best model; bootstrap validation confirmed its robustness (AUC = 0.940, 95% confidence interval: 0.891–0.977). A significant positive correlation was observed between the number of combined nuclei and XGBoost test AUC (Spearman's rho = 0.614, *P* < 0.001).

**Conclusion:**

A multi-nuclei QSM-based radiomics model integrating GP, RN, and Put with XGBoost achieves excellent diagnostic performance for PD, supporting the value of multi-regional information fusion in precision imaging-based diagnosis.

## Background

Parkinson's disease (PD) is the second most common neurodegenerative disorder worldwide and a leading cause of neurological disability (GBD 2021 Nervous System Disorders Collaborators, 2024). Its pathology is characterized by dopaminergic neuron loss in the substantia nigra pars compacta, accompanied by abnormal iron accumulation (Guo et al., 2025). Early and accurate diagnosis is essential for timely intervention and disease management, yet it remains clinically challenging. Current diagnostic criteria primarily rely on clinical manifestations, including bradykinesia, rigidity, and tremor, assessed using instruments such as the Unified Parkinson's Disease Rating Scale (UPDRS) (Postuma et al., 2015). However, these assessments are inherently subjective, and early-stage PD often presents with non-specific symptoms, leading to diagnostic uncertainty and delayed treatment (Tolosa et al., 2021). Conventional magnetic resonance imaging (MRI) lacks sufficient sensitivity to detect early microstructural changes in PD, underscoring the need for objective and quantitative imaging biomarkers (Heim et al., 2017).

Quantitative susceptibility mapping (QSM) quantifies tissue magnetic susceptibility and serves as a reliable measure of brain iron content (Chen et al., 2017). Accumulating evidence indicates that PD patients exhibit significantly elevated susceptibility values in deep gray matter nuclei, including the substantia nigra (SN), caudate nucleus (CN), globus pallidus (GP), red nucleus (RN), and putamen (Put), reflecting pathological iron deposition (Yang et al., 2022; Zeng et al., 2024; Prasuhn et al., 2022; Uchida et al., 2019). Nevertheless, most previous QSM studies have relied on conventional bulk susceptibility measurements, potentially overlooking subtle textural heterogeneity that may capture more complex pathological information (Fu et al., 2021). Radiomics enables high-throughput extraction of quantitative features that can detect subtle tissue alterations beyond the limits of visual perception (Scapicchio et al., 2021). When combined with machine learning algorithms, radiomics has shown considerable potential in disease diagnosis, prognosis prediction, and treatment response assessment across various neurological disorders (Chen et al., 2025b; Fathi Kazerooni et al., 2025). Several radiomics studies in PD using conventional MRI or QSM have reported encouraging diagnostic performance (Fu et al., 2025; Kang et al., 2022), though they have largely focused on single brain regions such as the SN alone, and whether integrating radiomic features from multiple subcortical nuclei could improve diagnostic accuracy remains to be systematically evaluated (Xiao et al., 2019).

Therefore, this study aimed to develop an accurate and interpretable QSM-based radiomics model for PD diagnosis by systematically assessing the diagnostic performance of radiomic features derived from different combinations of five subcortical nuclei.

## Methods

### Study population

**Figure 1:**
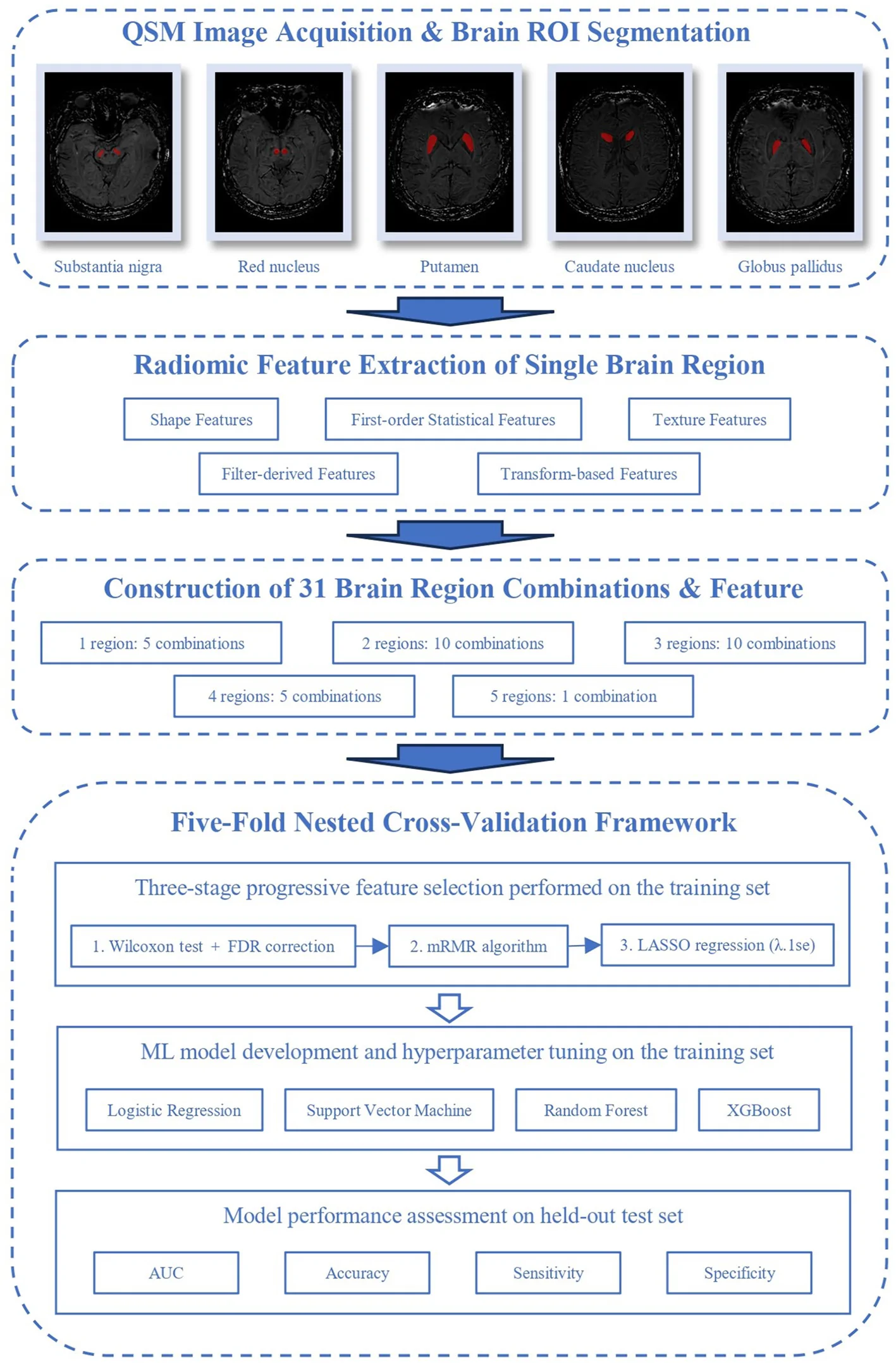
Study flowchart. QSM, quantitative susceptibility mapping; ROI, regions of interest; FDR, false discovery rate; mRMR, minimal redundancy maximal relevance; LASSO, least absolute shrinkage and selection operator; ML, machine learning; XGBoost, extreme gradient boosting; AUC, area under the receiver operating characteristic curve.

This study was approved by the Institutional Review Board of the local hospital (ethics approval No. 2025283) and was performed in accordance with the Declaration of Helsinki. Written informed consent was obtained from all participants. Patients with PD and healthy controls (HC) were prospectively recruited from the Department of Neurology between March 2024 and December 2025. PD was diagnosed by experienced neurologists using the UK Parkinson's Disease Society Brain Bank criteria (Greenland et al., 2026). Eligible participants were enrolled based on predefined inclusion and exclusion criteria, and demographic and clinical data—including age, sex, UPDRS score, and Hoehn and Yahr (H-Y) stage—were collected via standardized questionnaires. Exclusion criteria comprised: (1) secondary parkinsonism or atypical parkinsonian syndromes; (2) history of stroke, brain surgery, head trauma, or other neurological disorders affecting the basal ganglia or brainstem; (3) brain tumor or cerebral inflammatory diseases; (4) contraindications to MRI examination; (5) significant image artifacts on QSM; and (6) incomplete clinical or imaging data. HCs were recruited from the local community and met the same exclusion criteria, with no history of neurological or psychiatric disorders. Following eligibility screening, a total of 59 PD patients and 73 HCs were included. The study flowchart is shown in Figure 1.

### Image acquisition and region of interest segmentation

MRI was performed on a 3.0-T scanner (Ingenia Elition, Philips Healthcare, Best, The Netherlands) with a 32-channel head coil. QSM images were reconstructed from a 3D multi-echo gradient-echo sequence (10 echoes) using the following parameters: repetition time = 43 ms; first echo time = 4.1 ms; echo spacing = 4.1 ms; flip angle = 20°; pixel bandwidth = 775 Hz; field of view = 256 × 256 × 256 mm^3^; matrix size = 256 × 254 × 70; voxel size = 1 × 1 × 2 mm^3^; acquisition time = 6 min 22 s.

**Figure 2:**
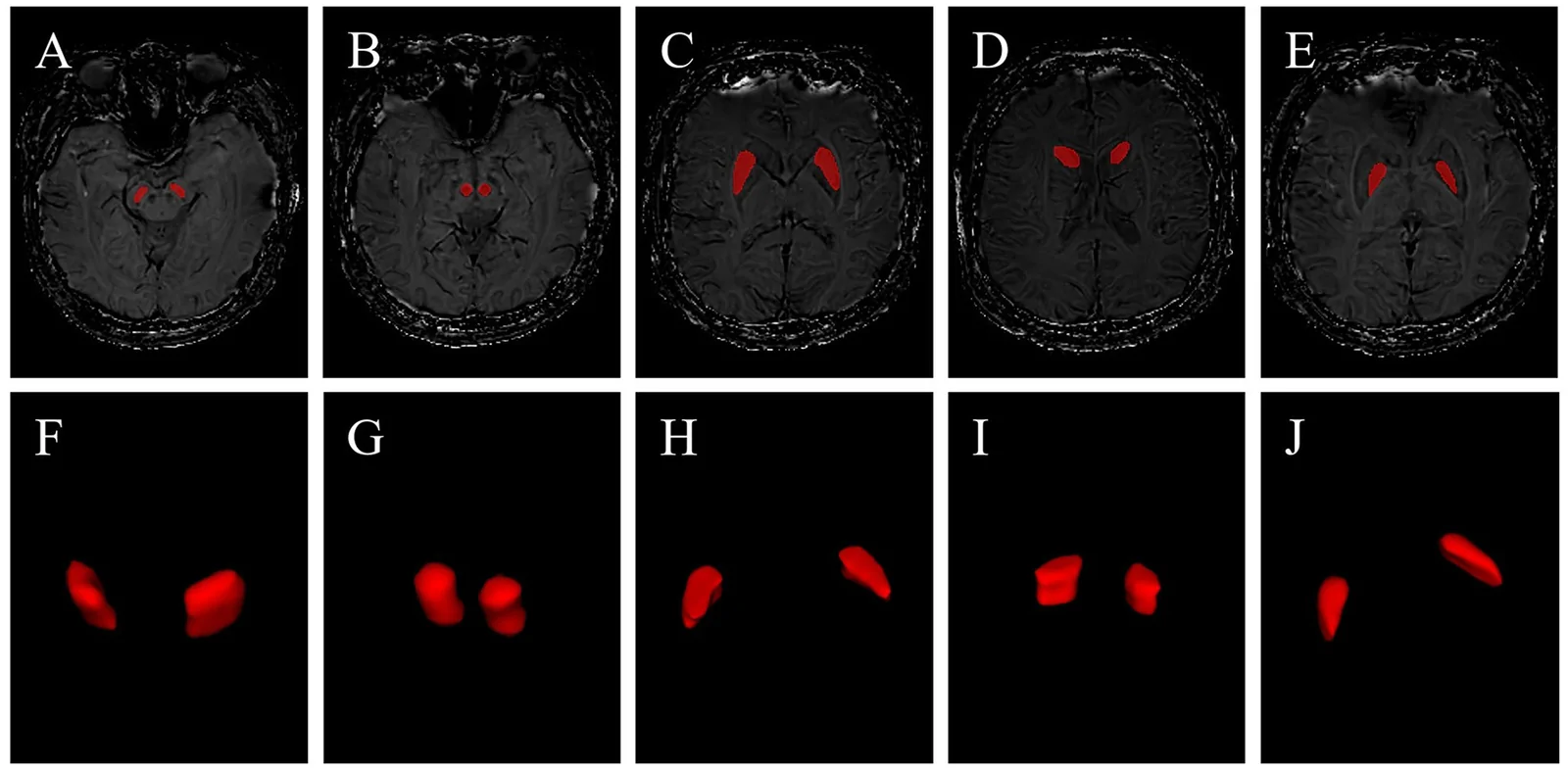
Quantitative susceptibility mapping images and regions of interest segmentation. **(A–J)** show 2D ROIs and 3D VOIs of the substantia nigra, red nucleus, putamen, caudate nucleus, and globus pallidus, which were used to extract radiomic features. First, 2D ROIs (approximately 3–5 layers per nucleus) were delineated for each participant **(A–E)**. Then, the 2D ROIs of the same brain region were merged into one 3D VOI. Finally, radiomic features were extracted from these 3D VOIs **(F–J)**. ROI, region of interest; VOI, volume of interest.

For each participant, five subcortical nuclei were manually delineated as regions of interest (ROIs) by an experienced neuroradiologist (with 8 years of experience) using 3D Slicer software (version 5.6.2) (Fedorov et al., 2012). ROIs were first drawn on consecutive 2D slices (~3–5 layers per nucleus) and then merged to form 3D volumes of interest (VOIs; Figure 2). The delineated structures included the SN, CN, GP, RN, and Put. To assess intra-rater reproducibility, a random subset of 20 participants was re-segmented by the same radiologist after a 2-week interval. For inter-rater reproducibility, a second experienced neuroradiologist independently performed ROI segmentation on the same subset using identical methods. Intra-class correlation coefficients (ICCs) were calculated for all extracted features; features with ICC > 0.75 were considered stable and retained for further analysis.

### Radiomic feature extraction and brain region combinations

Radiomic features were extracted from each 3D VOI using PyRadiomics (version 3.0.1) in Python 3.7 (van Griethuysen et al., 2017). For each brain region, features were derived from the original image and 13 derived image types: exponential, gradient, logarithm, square, square-root, and eight wavelet decompositions (LLH, LHL, LHH, HLL, HLH, HHL, HHH, LLL). The extracted features comprised first-order statistics (18 features), shape-based features (14 features, original image only), and texture features from five matrix types: gray-level co-occurrence matrix (GLCM, 24 features), gray-level run length matrix (GLRLM, 16 features), gray-level size zone matrix (GLSZM, 16 features), gray-level dependence matrix (GLDM, 14 features), and neighboring gray-tone difference matrix (NGTDM, 5 features). In total, 1,316 features were extracted per ROI.

To evaluate the predictive value of different subcortical structures, all 31 possible combinations of the five regions (SN, CN, GP, RN, Put) were generated, including 5 single regions, 10 pairs, 10 triples, 5 quadruples, and 1 quintuple. For each combination, radiomic features from the constituent regions were merged into a single candidate feature set. Additionally, a baseline model was constructed for each combination using only the mean susceptibility value of each constituent brain region as the sole input feature.

### Nested cross-validation framework

A stratified five-fold nested cross-validation framework was employed. In each outer iteration, four folds were used as the training set, and the remaining fold served as the test set, which was withheld from all feature selection, hyperparameter tuning, and model training. All model development procedures, including feature selection and hyperparameter optimization via an inner five-fold cross-validation, were performed exclusively on the training set. This yielded approximately 105–106 subjects for training and 26–27 for testing per outer fold, while the inner five-fold cross-validation further divided the training set into stratified subsets (~84–85 for inner training and 21–22 for inner validation).

### Feature selection

For each of the 31 brain-region combinations, a three-step feature selection procedure was performed independently within the training portion of each of the five outer cross-validation folds. First, features with a false discovery rate-adjusted *P* < 0.05 based on Wilcoxon rank-sum tests were retained. If more than 50 features remained, the top 50 were selected via minimal redundancy maximal relevance (mRMR). Finally, logistic regression with least absolute shrinkage and selection operator (LASSO) regularization and five-fold internal cross-validation was applied, retaining features with non-zero coefficients at the optimal penalty parameter λ (lambda.1se). This process yielded a unique feature subset for each of the 31 brain region combinations.

### Model development and hyperparameter tuning

Four classifiers were evaluated for each brain-region combination using the selected radiomic features. Within each training set, hyperparameters were optimized via inner five-fold cross-validation as described below. A logistic regression (LR) model with a binomial distribution was fitted using the selected features. A support vector machine (SVM) with a radial basis function kernel was employed; features were standardized to zero mean and unit variance with scaling parameters derived from the training set, and the regularization parameter *C* and kernel width σ were optimized simultaneously via five-fold cross-validation. A random forest (RF) model comprising 500 decision trees was constructed, and the number of features randomly sampled at each split was tuned via five-fold cross-validation to maximize the area under the receiver operating characteristic curve (AUC). An extreme gradient boosting (XGBoost) classifier was built with hyperparameters optimized in three sequential stages with early stopping using five-fold cross-validation: (1) maximum tree depth and minimum child weight; (2) subsample ratio and column subsampling ratio; (3) learning rate. The objective function was set to binary logistic regression, and the minimum loss reduction required for a further partition was fixed at 0. For each combination–classifier pair, once features and hyperparameters were finalized within each outer-training set, a model was trained on the entire training set and applied once to the held-out test fold to generate prediction probabilities. The baseline models based on mean susceptibility values were subjected to the same nested cross-validation framework and the same four classifiers.

### Model evaluation

For each combination–classifier pair, the AUC was computed, and accuracy, sensitivity, and specificity were computed at a threshold of 0.5 in each of the five test folds. The mean and standard deviation of these metrics across folds were then reported. The combination–classifier pair with the highest mean test-fold AUC was designated as the global best model. For this global best model, the receiver operating characteristic curve (ROC) was plotted using the out-of-fold predictions, decision curve analysis (DCA) was performed to evaluate the clinical net benefit across a range of threshold probabilities, and Shapley additive explanations (SHAP) values were computed to assess the contribution of each selected radiomic feature to the model output.

### Bootstrap stability analysis for the global best model

A bootstrap analysis was performed on the global best model. One thousand stratified bootstrap samples were drawn from the original dataset. For each bootstrap sample, the complete Stratified nested cross-validation pipeline was run identically: outer 5-fold cross-validation, with inner-loop feature selection and hyperparameter tuning. The out-of-fold AUC, accuracy, sensitivity, and specificity were recorded, and 95% confidence intervals (CI) were derived. The selection frequency of each radiomic feature was computed as the number of times the feature was retained by the LASSO step across all bootstrap runs.

### Statistical analysis

Baseline continuous variables were confirmed to be normally distributed using the Shapiro–Wilk test and are expressed as mean ± standard deviation. Between-group comparisons for continuous variables were performed using the independent samples *t*-test. Categorical variables are presented as frequencies (percentages) and were analyzed using the chi-square test. Spearman's rank correlation was used to examine the relationship between the number of combined brain regions and the mean test AUC of XGBoost models. Distributions of mean susceptibility values in each subcortical nucleus and of the top discriminative radiomic features were compared between PD and HC groups using box plots, and group differences were assessed with the Wilcoxon rank-sum test. All statistical tests were two-sided, and a *P* value < 0.05 was considered statistically significant. All analyses were performed using R software (version 4.2.2).

## Results

### Baseline characteristics

**Figure 3:**
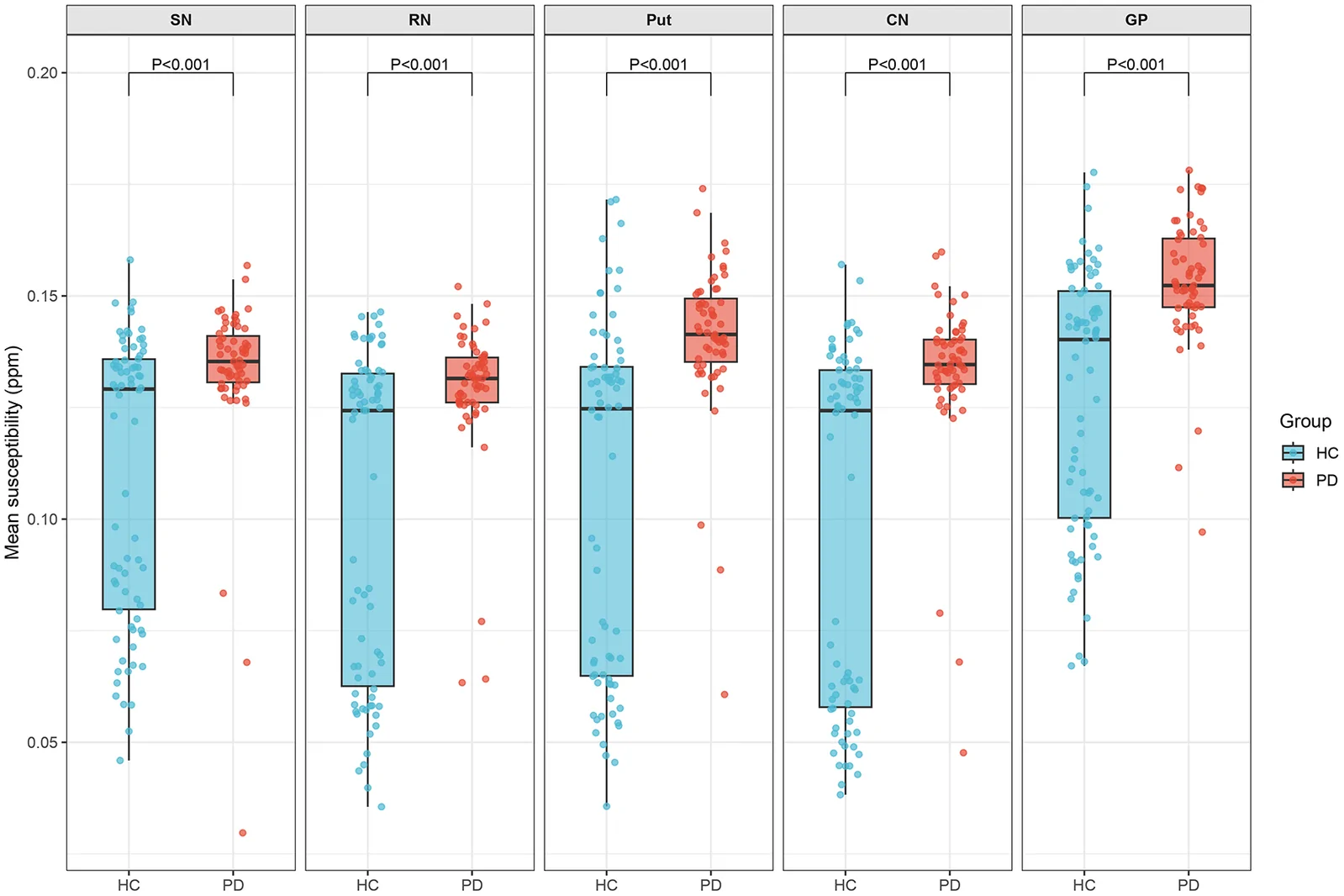
Comparison of QSM-derived mean susceptibility values across basal ganglia regions between HC and PD patients. *P*-values were derived from two-sided Wilcoxon rank-sum tests. QSM, quantitative susceptibility mapping; PD, Parkinson's disease; HC, healthy controls; SN, substantia nigra; RN, red nucleus; Put, putamen; CN, caudate nucleus; GP, globus pallidus.

No significant differences in age or sex were observed between PD patients and HCs (*P* > 0.05; Table 1). Comparisons of QSM-derived mean susceptibility values across the five subcortical nuclei are shown in Figure 3. PD patients exhibited significantly higher susceptibility values in all five nuclei compared with HCs (all *P* < 0.001).

**Table 1:** Baseline characteristics of participants.

Variable	PD (*n* = 59)	HC (*n* = 73)	*P*
Age (years), mean ± SD	63.51 ± 10.20	62.92 ± 13.19	0.772
Sex, *n* (%)			0.427
Male	26 (44.1%)	24 (32.9%)	
Female	33 (55.9%)	49 (67.1%)	
UPDRS score, mean ± SD	27.93 ± 24.02	–	–
H-Y stage, mean ± SD	1.80 ± 1.23	–	–

Data are presented as mean ± standard deviation or number (percentage). *P*-values for continuous variables were calculated using the independent samples *t*-test; for categorical variables, the chi-square test was used. PD, Parkinson's disease; HC, healthy controls; UPDRS, unified Parkinson's disease rating scale; H-Y, Hoehn and Yahr stage.

### Feature selection

Feature selection was performed independently for each of the 31 brain region combinations within each outer training fold of the nested cross-validation framework. The selection frequency of each radiomic feature for each brain region combination is provided in Additional File 1.

### Model performance and optimal brain region combinations

**Figure 4:**
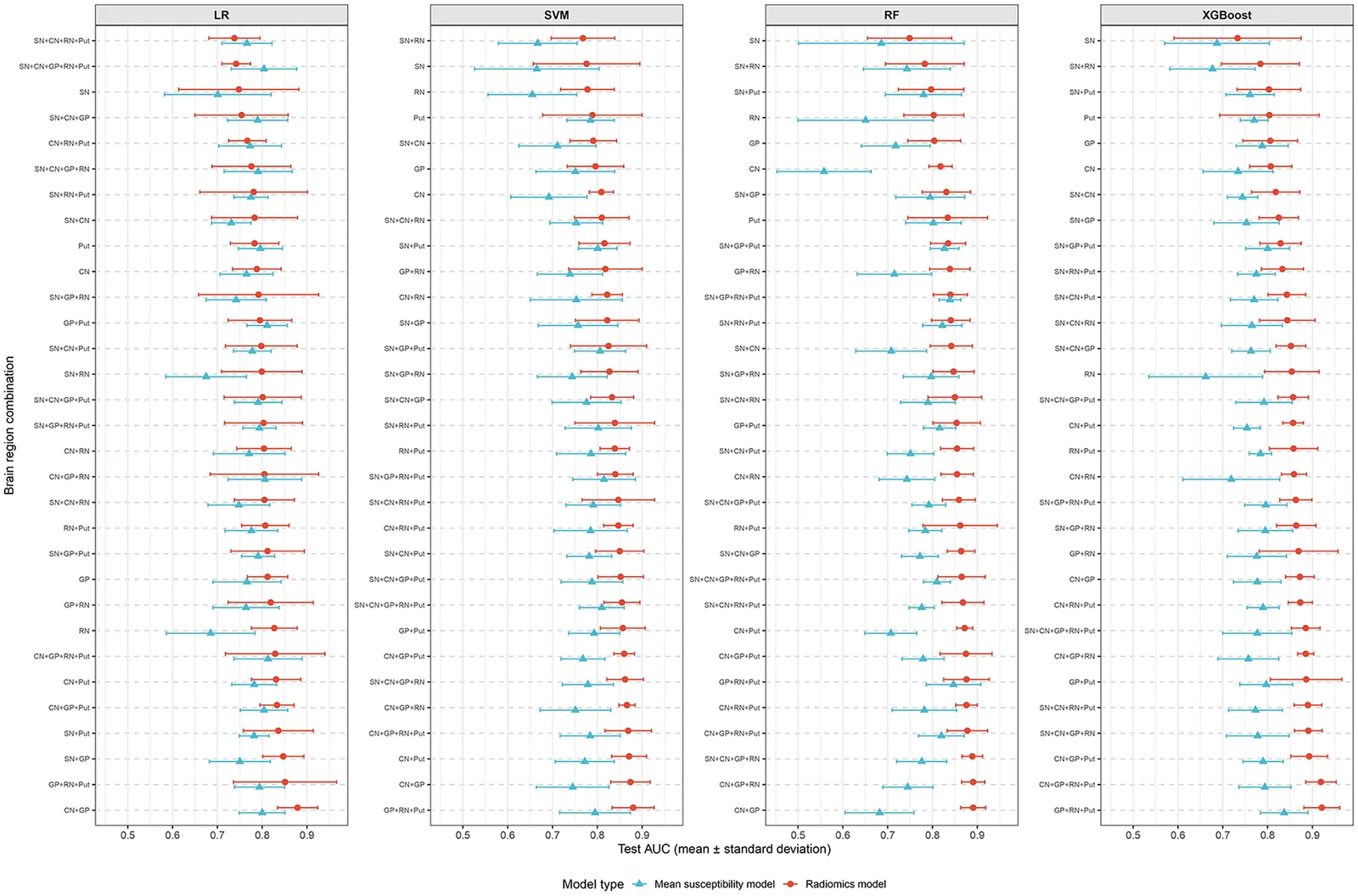
Comparison of test AUC between radiomics models and mean susceptibility models across different brain region combinations for four classifiers. Within each panel, combinations are ordered by descending radiomics test AUC. AUC, area under the receiver operating characteristic curve; LR, logistic regression; SVM, support vector machine; RF, random forest; XGBoost, extreme gradient boosting; SN, substantia nigra; CN, caudate nucleus; GP, globus pallidus; RN, red nucleus; Put, putamen.

The complete performance metrics for baseline models constructed using only the mean susceptibility values of the constituent nuclei are provided in Additional File 2 (Supplementary Tables S1–S4). The corresponding metrics for the radiomics-based models are presented in Additional File 2 (Supplementary Tables S5–S8). A forest plot comparing the mean test AUCs of the radiomics models and the baseline models across all 31 combinations and four classifiers is shown in Figure 4. Radiomics models consistently yielded higher mean test AUCs than their baseline counterparts, confirming the incremental diagnostic value of radiomic texture features over simple mean susceptibility measurements.

Among the radiomics models, LR achieved its best performance with CN + GP, yielding a mean test AUC of 0.879 ± 0.045 (Additional File 2, Supplementary Table S5). SVM attained its highest mean test AUC using GP + RN + Put, with a mean test AUC of 0.880 ± 0.047 (Additional File 2, Supplementary Table S6). RF performed best with CN + GP, achieving a mean test AUC of 0.891 ± 0.028 (Additional File 2, Supplementary Table S7). XGBoost with GP + RN + Put produced the highest mean test AUC among all evaluated models (0.921 ± 0.040; Additional File 2, Supplementary Table S8) and was designated the global best model.

### Detailed evaluation and feature interpretation of the global best model

**Figure 5:**
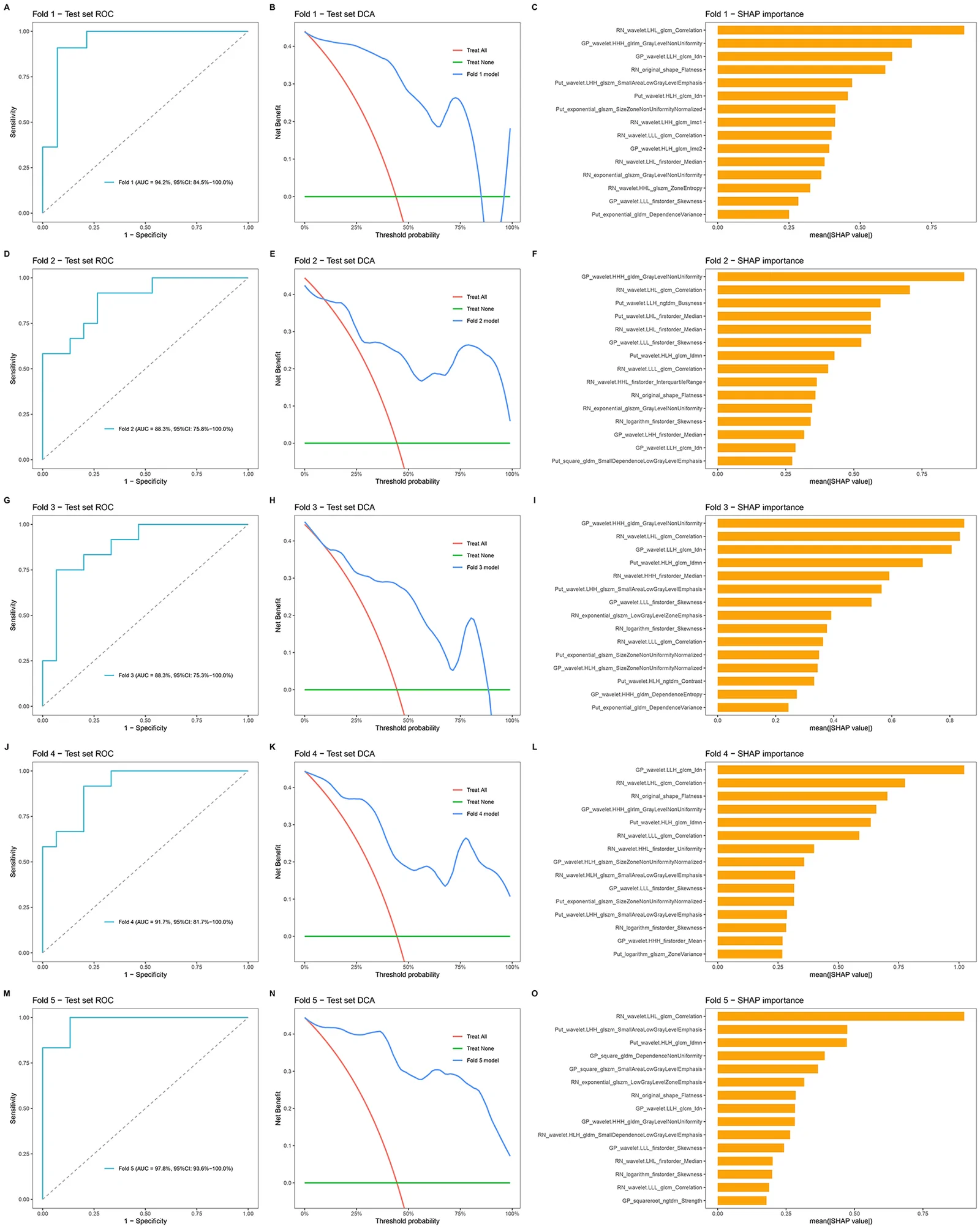
Five-fold cross-validation performance and feature importance of the GP + RN + Put XGBoost model. Panels are arranged in five rows (Fold 1 to Fold 5) and three columns. Within each row, the left panel shows the ROC curve of the test set, with AUC and 95% CI given in the legend **(A, D, G, J, M)**. The middle panel displays the DCA of the same test set **(B, E, H, K, N)**. The right panel presents the top 15 most important features according to mean absolute SHAP value, derived from the corresponding training set **(C, F, I, L, O)**. GP, globus pallidus; RN, red nucleus; Put: putamen; XGBoost, extreme gradient boosting; ROC, receiver operating characteristic; AUC, area under the ROC curve; CI, confidence interval; DCA, decision curve analysis; SHAP, Shapley additive explanations.

Using a classification threshold of 0.5, the global best model (XGBoost with GP + RN + Put) achieved a mean accuracy of 0.805 ± 0.046, sensitivity of 0.798 ± 0.093, and specificity of 0.810 ± 0.032 across the five test folds (Additional File 2, Supplementary Table S8). The per-fold ROC curves, DCA, and SHAP feature importance plots are presented in Figure 5. The ROC curves demonstrated consistently high discriminatory performance across all folds. DCA for each fold indicated a favorable clinical net benefit across all threshold probabilities, outperforming both the “treat all” and “treat none” strategies. SHAP analysis revealed that the most influential feature varied across folds: RN_wavelet.LHL_glcm_Correlation ranked highest in two-folds, GP_wavelet.HHH_gldm_GrayLevelNonUniformity ranked highest in two-folds, and GP_wavelet.LLH_glcm_Idn ranked highest in one-fold.

### Impact of brain region number on model performance

**Figure 6:**
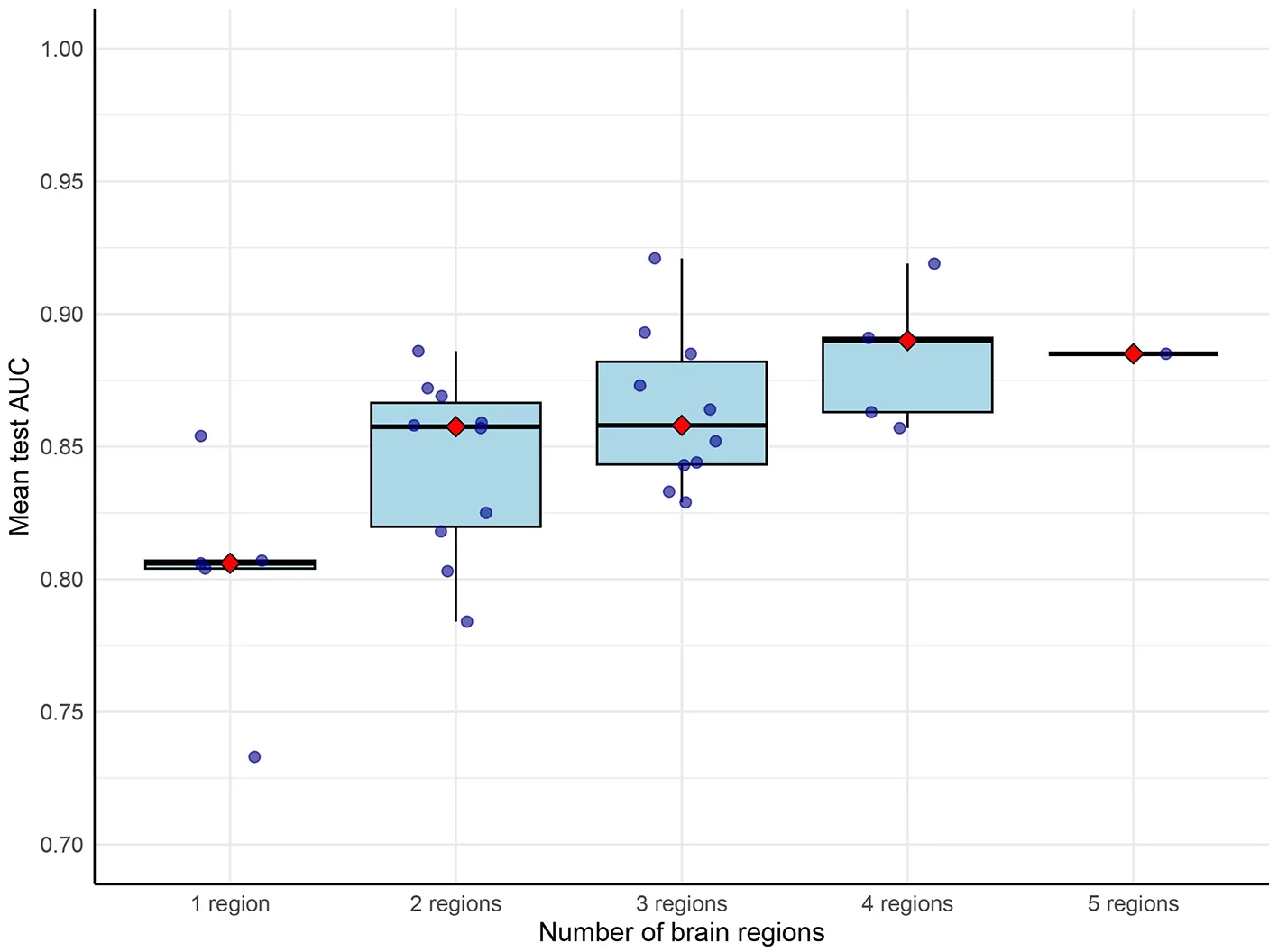
Effect of the number of combined brain regions on the cross-validated test AUC of the XGBoost radiomics model. AUC, area under the receiver operating characteristic curve; XGBoost, extreme gradient boosting.

Spearman correlation revealed a significant positive relationship between the number of combined brain regions and the mean test AUC for XGBoost models across all 31 combinations (rho = 0.614, *P* < 0.001), indicating that incorporating more regions tends to improve predictive performance (Figure 6).

### Bootstrap stability analysis

**Figure 7:**
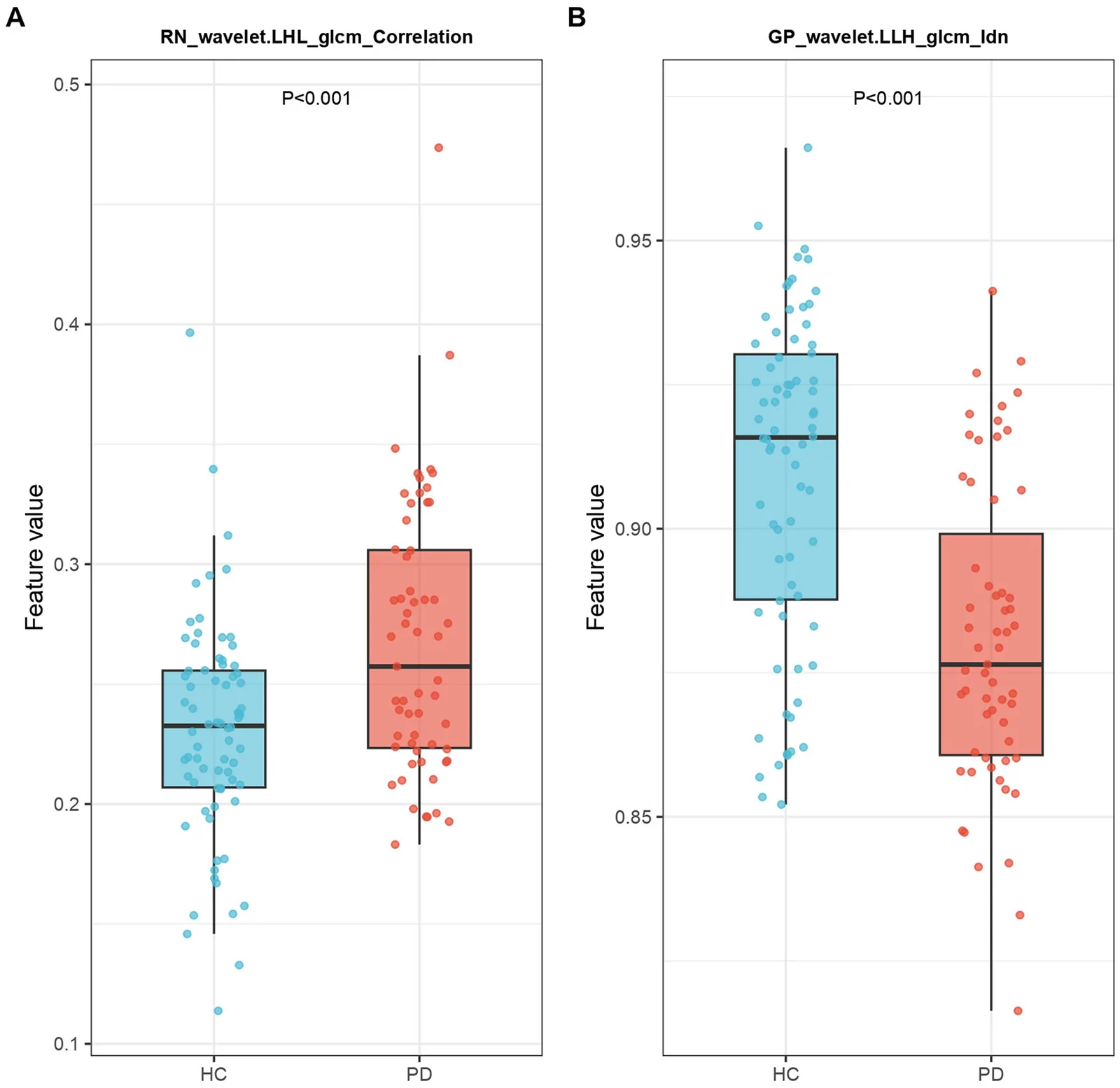
Stable radiomic features with high bootstrap selection frequency for discriminating PD from HC. Boxplots of two robust features that survived >80 % of 1,000 bootstrap iterations with nested cross-validation. **(A)** Median RN_wavelet.LHL_glcm_Correlation was higher in PD than in HC. **(B)** Median GP_wavelet.LLH_glcm_Idn was higher in HC than in PD. *P*-values were derived from two-sided Wilcoxon rank-sum tests. GP, globus pallidus; RN, red nucleus; PD, Parkinson's disease; HC, healthy control.

One thousand stratified bootstrap iterations of the nested cross-validation pipeline were performed for the optimal GP + RN + Put XGBoost model. The bootstrap-estimated AUC was 0.940 (95% CI: 0.891–0.977), with an accuracy of 0.879 (95% CI: 0.813–0.932), sensitivity of 0.859 (95% CI: 0.762–0.948), and specificity of 0.895 (95% CI: 0.824–0.959; Table 2). Radiomic features that survived the LASSO step in more than 80% of the bootstrap iterations were considered robust. Two such features were identified: RN_wavelet.LHL_glcm_Correlation and GP_wavelet.LLH_glcm_Idn. Box plots comparing the distributions of these two features between PD and HC groups are shown in Figure 7, with all comparisons reaching statistical significance (*P* < 0.001). The detailed per-iteration performance metrics and feature selection frequencies across the 1,000 bootstrap replicates are provided in Additional File 3.

**Table 2:** Bootstrap stability analysis of the optimal radiomics model (GP + RN + Put, XGBoost).

Metric	Mean	SD	95% CI
AUC	0.940	0.022	0.891–0.977
Accuracy	0.879	0.030	0.813–0.932
Sensitivity	0.859	0.047	0.762–0.948
Specificity	0.895	0.035	0.824–0.959

Performance metrics were estimated from 1,000 bootstrap replicates, each involving resampling followed by nested 5-fold cross-validation. Mean, SD, and 95% CI are reported. The 95% CI was obtained using the bootstrap percentile method (2.5th and 97.5th percentiles of the bootstrap distribution). GP, globus pallidus; RN, red nucleus; Put, putamen; XGBoost, extreme gradient boosting; AUC, area under the receiver operating characteristic curve; SD, standard deviation; CI, confidence interval.

## Discussion

In this study, we systematically evaluated all possible combinations of radiomic features derived from QSM of five subcortical nuclei for PD diagnosis using four machine learning classifiers under a rigorous nested cross-validation framework. The key finding is that a multi-nuclei integration strategy substantially improves diagnostic performance compared to single-region approaches. Specifically, the XGBoost classifier combining the GP, RN, and Put achieved the highest cross-validated diagnostic accuracy, with a bootstrap-estimated AUC of 0.940, highlighting the value of fusing complementary pathological information from interconnected nodes of the basal ganglia–thalamocortical motor circuit. These results provide robust internal validation evidence that spatially distributed iron deposition and microstructural alterations—captured by high-dimensional radiomic texture features—collectively contribute to the imaging-based differentiation of PD from healthy aging.

Notably, the optimal combination did not include the SN, the traditional core pathological region of PD (Yao et al., 2024). This observation should be interpreted with caution rather than as a definitive medical conclusion. Indeed, although mean SN susceptibility was significantly elevated in PD patients, radiomic texture features—which primarily reflect spatial heterogeneity as opposed to average iron content—are distinct from bulk measurements and may be less discriminative when derived from the whole SN, notably because iron deposition in this nucleus is highly focal and predominantly affects the nigrosome-1 subregion (Mueller et al., 2014; Bergsland et al., 2019). Importantly, this finding does not contradict the established pathological role of the SN; rather, it suggests that microstructural alterations captured by radiomic features in the GP, RN, and Put may provide more statistically robust signatures for classification in this dataset. However, alternative explanations involving manual segmentation variability, limited sample size, and feature-selection instability cannot be ruled out. We therefore regard this observation as hypothesis-generating. Moreover, a significant positive correlation was observed between the number of combined nuclei and the cross-validated test AUC of XGBoost models (rho = 0.614, *P* < 0.001), supporting the diffuse involvement of multiple subcortical structures in PD pathology (Jin et al., 2025; Ren et al., 2026). The marginal performance improvement of four- or five-nuclei combinations over three-nuclei combinations suggests potential feature redundancy with excessive regional inclusion, providing a practical basis for rational selection of nuclei in future radiomic studies.

The principal innovation of this study lies in the first systematic comparison of all possible multi-nuclei combinations of QSM-based radiomic features for PD diagnosis, addressing a critical gap in previous studies that predominantly focused on single regions. Radiomics models consistently outperformed baseline models constructed solely from mean susceptibility values, confirming the incremental diagnostic value of texture features beyond conventional bulk measurements. The observed performance advantage is likely attributable to three factors: the integration of complementary pathological information from interconnected motor circuit nuclei, the comprehensive feature extraction pipeline incorporating original and 13 derived image types, and the capacity of machine learning classifiers to capture complex non-linear interactions among high-dimensional radiomic features (Chen et al., 2025a; Xiong et al., 2026).

A direct numerical comparison with existing QSM-based radiomics studies warrants caution due to substantial methodological heterogeneity. Xiao et al. reported an AUC of 0.89 with logistic regression and SVM classifiers applied to features from the left and right SN under nested cross-validation, providing a rigorous internal validation benchmark (Xiao et al., 2019). Cheng et al. achieved an AUC of 0.96 using SVM on SN features that included the nigrosome-1 subregion; however, feature selection was performed on the entire dataset before cross-validation, potentially introducing optimistic bias (Cheng et al., 2019; Demircioglu, 2021). Kang et al. reported an AUC of 0.95 using SVM on SN, caudate head, and Put features; however, their reliance on a single train-test split in a modestly sized cohort may yield less generalizable performance estimates (Kang et al., 2022; Singh et al., 2021). Several key differences must be considered: nested cross-validation yields more conservative estimates than single-split or whole-dataset feature selection approaches; ROI definition varies across studies; and sample sizes, feature extraction pipelines, and classifiers differ considerably. Our model achieved a bootstrap-estimated AUC of 0.940 under nested cross-validation, comparing favorably with the rigorously validated estimate of Xiao et al. while acknowledging that differences in the number and identity of included nuclei preclude a strictly controlled comparison (Xiao et al., 2019). The observation that multi-nuclei models perform well—both in our systematic evaluation and in the three-region analysis of Kang et al.—provides converging evidence that iron-related microstructural alterations in PD extend beyond the SN to involve interconnected basal ganglia structures (Kang et al., 2022). Nevertheless, all findings should be regarded as hypothesis-generating until independently validated in large-scale multicenter cohorts.

Among the four classifiers evaluated, ensemble learning methods—RF and XGBoost—demonstrated numerically higher cross-validated AUCs relative to conventional single learners, LR and SVM, across the majority of region combinations. This pattern is consistent with the high-dimensional, intercorrelated nature of radiomic data, in which tree-based ensemble methods can effectively capture complex non-linear feature interactions without requiring explicit specification of interaction terms. Notably, XGBoost achieved the highest mean cross-validated AUC of 0.921 ± 0.040 among all evaluated models, a numerical advantage attributable to its adaptive regularization, early stopping strategy, and gradient-based optimization, which mitigate overfitting while capturing subtle higher-order patterns in high-dimensional radiomic data (Chen and Guestrin, 2016). Formal statistical testing of pairwise classifier comparisons is warranted in future studies with larger samples.

Beyond predictive accuracy, we employed SHAP analysis to bridge the gap between high-dimensional radiomic features and underlying neuropathology (Lundberg et al., 2020). Across the five cross-validation folds, three features consistently emerged as the most influential: RN_wavelet.LHL_glcm_Correlation (ranked first in two folds), GP_wavelet.HHH_gldm_GrayLevelNonUniformity (ranked first in two folds) and GP_wavelet.LLH_glcm_Idn (ranked first in one fold). Notably, two of these—RN_wavelet.LHL_glcm_Correlation and GP_wavelet.LLH_glcm_Idn—were also the only features that survived LASSO selection in more than 80% of the 1,000 bootstrap iterations, demonstrating that the features most critical to model decision-making are also the most robust to sampling variability. Both were derived from wavelet-transformed images, underscoring the value of multiscale decomposition (van Griethuysen et al., 2017). RN_wavelet.LHL_glcm_Correlation, a GLCM texture metric from the RN, showed higher values in PD, indicating more homogeneous local susceptibility patterns consistent with spatially organized pathological iron deposition (Jin et al., 2025). GP_wavelet.LLH_glcm_Idn from the GP showed lower values in PD, reflecting disrupted tissue uniformity likely attributable to spatially heterogeneous iron accumulation and reactive gliosis (McNamara et al., 2026). These two robust features—one reflecting increased textural order in the RN and the other reflecting decreased homogeneity in the GP—highlight the complementary nature of multi-nuclei radiomic profiles in capturing distinct aspects of PD-related microstructural pathology.

## Limitations

Several limitations warrant consideration. First, this was a single-center study with a relatively limited sample size and no independent external validation cohort. Although we employed nested cross-validation and bootstrap stability analyses to obtain less biased performance estimates, these internal procedures cannot substitute for external validation across different scanners, field strengths, and acquisition protocols. To the best of our knowledge, no large-scale public neuroimaging database currently provides ready-to-use QSM images for Parkinson's disease, highlighting the need for multicenter prospective cohorts. Second, ROI segmentation relied on manual delineation. Although we applied ICC-based feature filtering to ensure reproducibility, manual delineation remains labor-intensive and may introduce operator-dependent variability that affects texture features. Automated segmentation tools specifically developed for QSM data are currently lacking and warrant future investigation. Third, several clinically relevant variables—including disease duration, levodopa equivalent daily dose, medication status at the time of scanning, and formal motor phenotype classification—were not systematically recorded, precluding adjustment for potential confounders. Healthy controls did not undergo UPDRS assessment, which prevented the construction of a clinical-demographic baseline model for comparison. Fourth, feature selection exhibited some instability across cross-validation folds and bootstrap replicates, reflecting the high dimensionality of radiomic data and the limited sample size. Formal correction for multiple testing across the 124 evaluated pipelines was not performed, as our primary aim was to transparently report comparative performance; consequently, the observed model ranking should be interpreted with appropriate caution.

## Conclusions

This study demonstrates that a multi-nuclei radiomics approach based on QSM, combined with ensemble machine learning, achieves excellent diagnostic performance for PD. The XGBoost model integrating the GP, RN, and Put provided the highest accuracy among the evaluated strategies, underscoring the value of multi-regional information fusion and offering an objective, interpretable framework for precision imaging-based diagnosis of PD.

## Data Availability

The raw data supporting the conclusions of this article will be made available by the authors, without undue reservation.
