# Supplementary material for: Machine learning-based radiomics modeling of combined subcortical nuclei from quantitative susceptibility mapping for Parkinson's disease diagnosis
Source: Front Aging Neurosci. 2026 Aug 11;18:1869390. doi: 10.3389/fnagi.2026.1869390 (PMC13503537; doi:10.3389/fnagi.2026.1869390)
Supplement: Supplementary file 1 [file Data_Sheet_1.pdf]

Table S1. Performance of logistic regression models using mean susceptibility from different brain region combinations with 5-fold cross-validation.

Brain Region Combination	AUC	Accuracy	Sensitivity	Specificity
CN+GP+RN+Put	0.813±0.076	0.752±0.048	0.798±0.138	0.715±0.062
GP+Put	0.811±0.045	0.782±0.028	0.883±0.112	0.702±0.093
CN+GP+RN	0.806±0.082	0.753±0.053	0.797±0.139	0.717±0.051
CN+GP+Put	0.804±0.053	0.767±0.045	0.917±0.083	0.649±0.055
SN+CN+GP+RN+Put	0.804±0.073	0.761±0.063	0.798±0.138	0.730±0.079
CN+GP	0.800±0.051	0.743±0.040	0.898±0.069	0.620±0.087
Put	0.796±0.049	0.737±0.009	0.817±0.124	0.673±0.112
GP+RN+Put	0.794±0.056	0.759±0.046	0.848±0.108	0.688±0.108
SN+GP+RN+Put	0.794±0.037	0.752±0.055	0.832±0.081	0.689±0.103
SN+GP+Put	0.791±0.037	0.760±0.059	0.850±0.109	0.689±0.103
SN+CN+GP+RN	0.791±0.076	0.760±0.059	0.797±0.151	0.730±0.042
SN+CN+GP+Put	0.791±0.053	0.760±0.038	0.848±0.068	0.690±0.057
SN+CN+GP	0.790±0.067	0.752±0.031	0.830±0.059	0.690±0.057
SN+Put	0.782±0.033	0.768±0.057	0.800±0.151	0.743±0.058
CN+Put	0.782±0.050	0.707±0.027	0.833±0.102	0.607±0.095
SN+CN+Put	0.778±0.042	0.746±0.056	0.817±0.149	0.690±0.057

Brain Region Combination	AUC	Accuracy	Sensitivity	Specificity
RN+Put	0.776±0.059	0.737±0.020	0.782±0.110	0.702±0.064
SN+RN+Put	0.775±0.038	0.745±0.028	0.765±0.106	0.729±0.073
CN+RN+Put	0.773±0.070	0.738±0.066	0.748±0.129	0.730±0.048
CN+RN	0.771±0.080	0.677±0.048	0.765±0.147	0.607±0.095
GP	0.766±0.076	0.737±0.059	0.762±0.150	0.716±0.087
SN+CN+RN+Put	0.766±0.056	0.723±0.063	0.748±0.129	0.703±0.058
CN	0.765±0.059	0.699±0.047	0.883±0.075	0.552±0.111
GP+RN	0.764±0.074	0.690±0.083	0.779±0.153	0.620±0.087
SN+GP	0.750±0.068	0.713±0.086	0.779±0.153	0.660±0.134
SN+CN+RN	0.748±0.069	0.677±0.066	0.765±0.147	0.607±0.134
SN+GP+RN	0.742±0.067	0.713±0.078	0.795±0.129	0.647±0.119
SN+CN	0.731±0.044	0.684±0.065	0.867±0.095	0.538±0.133
SN	0.701±0.119	0.652±0.096	0.798±0.125	0.536±0.205
RN	0.685±0.099	0.653±0.084	0.814±0.069	0.525±0.145
SN+RN	0.675±0.090	0.660±0.083	0.865±0.044	0.497±0.161

Note: Values are presented as mean ± standard deviation across 5 folds. Brain region combinations were ranked by descending mean validation AUC.

Abbreviations: AUC, area under the receiver operating characteristic curve; SN, substantia nigra; GP, globus pallidus; CN, caudate nucleus; Put, putamen; RN, red nucleus.

Table S2. Performance of Support Vector Machine models using mean susceptibility from different brain region combinations with 5-fold cross-validation.

Brain Region Combination	AUC	Accuracy	Sensitivity	Specificity
SN+GP+RN+Put	0.815±0.070	0.715±0.025	0.682±0.178	0.742±0.114
SN+CN+GP+RN+Put	0.810±0.050	0.715±0.036	0.698±0.149	0.729±0.119
SN+GP+Put	0.806±0.057	0.700±0.046	0.682±0.158	0.715±0.091
SN+RN+Put	0.802±0.074	0.716±0.072	0.632±0.180	0.784±0.055
SN+Put	0.801±0.043	0.739±0.083	0.683±0.190	0.784±0.055
GP+RN+Put	0.795±0.079	0.730±0.042	0.697±0.123	0.757±0.074
GP+Put	0.793±0.057	0.730±0.056	0.645±0.104	0.798±0.064
SN+CN+RN+Put	0.791±0.061	0.732±0.085	0.648±0.178	0.798±0.064
SN+CN+GP+Put	0.788±0.069	0.701±0.073	0.683±0.199	0.715±0.091
RN+Put	0.786±0.077	0.746±0.076	0.665±0.164	0.811±0.054
Put	0.785±0.053	0.760±0.028	0.697±0.068	0.810±0.032
CN+RN+Put	0.785±0.082	0.732±0.099	0.648±0.178	0.798±0.064
CN+GP+RN+Put	0.784±0.067	0.684±0.041	0.629±0.121	0.729±0.087
SN+CN+Put	0.782±0.050	0.724±0.067	0.715±0.108	0.730±0.042
SN+CN+GP+RN	0.779±0.057	0.708±0.033	0.665±0.184	0.742±0.114
SN+CN+GP	0.776±0.077	0.692±0.031	0.715±0.160	0.673±0.112

Brain Region Combination	AUC	Accuracy	Sensitivity	Specificity
CN+Put	0.772±0.066	0.745±0.035	0.680±0.085	0.797±0.048
CN+GP+Put	0.768±0.049	0.722±0.046	0.712±0.043	0.730±0.063
SN+GP	0.757±0.089	0.693±0.041	0.700±0.192	0.688±0.108
CN+RN	0.753±0.103	0.716±0.081	0.614±0.120	0.798±0.064
SN+CN+RN	0.753±0.059	0.669±0.027	0.648±0.187	0.687±0.132
GP	0.751±0.088	0.692±0.058	0.679±0.135	0.703±0.058
CN+GP+RN	0.751±0.079	0.708±0.055	0.647±0.102	0.757±0.033
CN+GP	0.745±0.081	0.677±0.073	0.664±0.127	0.689±0.063
SN+GP+RN	0.744±0.078	0.692±0.086	0.765±0.179	0.634±0.065
GP+RN	0.739±0.073	0.677±0.080	0.645±0.104	0.702±0.104
SN+CN	0.711±0.086	0.676±0.069	0.697±0.123	0.659±0.169
CN	0.692±0.085	0.596±0.127	0.479±0.191	0.690±0.160
SN+RN	0.667±0.088	0.616±0.056	0.597±0.185	0.632±0.118
SN	0.665±0.139	0.579±0.082	0.667±0.333	0.540±0.230
RN	0.655±0.099	0.653±0.124	0.609±0.242	0.688±0.108

Note: Values are presented as mean ± standard deviation across 5 folds. Brain region combinations were ranked by descending mean validation AUC.

Abbreviations: AUC, area under the receiver operating characteristic curve; SN, substantia nigra; GP, globus pallidus; CN, caudate nucleus; Put, putamen; RN, red nucleus.

Table S3. Performance of Random Forest models using mean susceptibility from different brain region combinations with 5-fold cross-validation.

Brain Region Combination	AUC	Accuracy	Sensitivity	Specificity
GP+RN+Put	0.847±0.061	0.738±0.047	0.747±0.080	0.730±0.103
SN+GP+RN+Put	0.839±0.024	0.706±0.049	0.711±0.143	0.702±0.064
SN+GP+Put	0.827±0.032	0.766±0.037	0.744±0.092	0.784±0.055
SN+RN+Put	0.822±0.044	0.722±0.037	0.714±0.160	0.730±0.067
CN+GP+RN+Put	0.820±0.051	0.714±0.016	0.712±0.043	0.716±0.029
GP+Put	0.816±0.036	0.736±0.044	0.658±0.134	0.798±0.064
SN+CN+GP+RN+Put	0.810±0.030	0.752±0.017	0.764±0.066	0.743±0.058
Put	0.802±0.062	0.730±0.064	0.611±0.039	0.825±0.100
SN+GP+RN	0.797±0.062	0.721±0.087	0.709±0.208	0.730±0.067
SN+GP	0.795±0.077	0.722±0.085	0.677±0.200	0.757±0.088
SN+CN+GP+Put	0.792±0.038	0.706±0.034	0.677±0.073	0.730±0.009
SN+CN+RN	0.790±0.061	0.698±0.058	0.679±0.088	0.714±0.085
RN+Put	0.784±0.037	0.677±0.048	0.632±0.170	0.713±0.163
CN+RN+Put	0.782±0.072	0.693±0.102	0.647±0.144	0.730±0.103
SN+Put	0.780±0.085	0.729±0.098	0.677±0.172	0.770±0.063
CN+GP+Put	0.779±0.047	0.722±0.037	0.695±0.110	0.744±0.053

Brain Region Combination	AUC	Accuracy	Sensitivity	Specificity
SN+CN+GP+RN	0.776±0.056	0.723±0.057	0.695±0.093	0.745±0.084
SN+CN+RN+Put	0.776±0.028	0.669±0.027	0.629±0.121	0.702±0.080
SN+CN+GP	0.772±0.041	0.692±0.044	0.661±0.060	0.717±0.069
SN+CN+Put	0.751±0.052	0.690±0.053	0.694±0.053	0.688±0.085
CN+GP+RN	0.745±0.056	0.714±0.031	0.711±0.116	0.717±0.084
SN+RN	0.743±0.097	0.677±0.084	0.682±0.258	0.673±0.112
CN+RN	0.743±0.062	0.714±0.046	0.714±0.123	0.714±0.085
GP	0.718±0.077	0.608±0.055	0.591±0.146	0.622±0.031
GP+RN	0.715±0.083	0.646±0.076	0.626±0.176	0.662±0.048
SN+CN	0.708±0.079	0.684±0.056	0.695±0.071	0.675±0.075
CN+Put	0.707±0.058	0.637±0.071	0.627±0.042	0.646±0.134
SN	0.686±0.185	0.660±0.132	0.694±0.129	0.632±0.165
CN+GP	0.682±0.077	0.661±0.070	0.661±0.132	0.661±0.075
RN	0.651±0.151	0.602±0.097	0.545±0.177	0.648±0.132
CN	0.558±0.105	0.479±0.069	0.423±0.097	0.525±0.120

Note: Values are presented as mean ± standard deviation across 5 folds. Brain region combinations were ranked by descending mean validation AUC.

Abbreviations: AUC, area under the receiver operating characteristic curve; SN, substantia nigra; GP, globus pallidus; CN, caudate nucleus; Put, putamen; RN, red nucleus.

Table S4. Performance of XGBoost models using mean susceptibility from different brain region combinations with 5-fold cross-validation.

Brain Region Combination	AUC	Accuracy	Sensitivity	Specificity
GP+RN+Put	0.837±0.053	0.746±0.056	0.732±0.237	0.757±0.153
SN+GP+Put	0.800±0.049	0.723±0.100	0.809±0.104	0.730±0.049
GP+Put	0.797±0.059	0.693±0.091	0.705±0.218	0.746±0.112
SN+GP+RN+Put	0.796±0.047	0.720±0.069	0.761±0.100	0.688±0.097
SN+GP+RN	0.795±0.061	0.714±0.037	0.764±0.179	0.674±0.115
CN+GP+RN+Put	0.794±0.058	0.722±0.059	0.747±0.154	0.703±0.075
SN+CN+GP+Put	0.792±0.063	0.767±0.016	0.797±0.073	0.743±0.088
CN+GP+Put	0.790±0.045	0.730±0.103	0.788±0.081	0.763±0.065
CN+RN+Put	0.790±0.036	0.753±0.057	0.817±0.109	0.703±0.075
GP	0.788±0.058	0.744±0.067	0.762±0.181	0.730±0.106
RN+Put	0.784±0.025	0.761±0.044	0.833±0.132	0.703±0.075
SN+CN+GP+RN	0.778±0.070	0.670±0.056	0.662±0.114	0.676±0.084
CN+GP	0.777±0.053	0.700±0.033	0.712±0.094	0.690±0.074
SN+CN+GP+RN+Put	0.777±0.077	0.709±0.115	0.771±0.158	0.729±0.095
GP+RN	0.776±0.066	0.707±0.059	0.695±0.208	0.716±0.073
SN+RN+Put	0.775±0.042	0.713±0.044	0.814±0.091	0.633±0.088

Brain Region Combination	AUC	Accuracy	Sensitivity	Specificity
SN+CN+RN+Put	0.773±0.060	0.724±0.089	0.733±0.285	0.716±0.099
Put	0.770±0.031	0.716±0.103	0.812±0.142	0.712±0.031
SN+CN+Put	0.770±0.053	0.731±0.076	0.733±0.216	0.730±0.082
SN+CN+RN	0.765±0.068	0.683±0.075	0.712±0.094	0.660±0.150
SN+CN+GP	0.763±0.043	0.682±0.075	0.667±0.245	0.750±0.148
SN+Put	0.761±0.054	0.660±0.102	0.688±0.197	0.683±0.033
CN+GP+RN	0.757±0.068	0.700±0.056	0.662±0.114	0.730±0.048
CN+Put	0.754±0.030	0.716±0.103	0.833±0.136	0.695±0.084
SN+GP	0.753±0.073	0.689±0.130	0.779±0.153	0.617±0.211
SN+CN	0.744±0.034	0.685±0.083	0.765±0.083	0.679±0.146
CN	0.734±0.078	0.685±0.074	0.645±0.157	0.716±0.099
CN+RN	0.719±0.108	0.669±0.083	0.695±0.125	0.649±0.128
SN	0.687±0.117	0.585±0.109	0.662±0.284	0.562±0.241
SN+RN	0.677±0.095	0.615±0.119	0.798±0.103	0.540±0.230
RN	0.662±0.127	0.602±0.072	0.617±0.247	0.591±0.206

Note: Values are presented as mean ± standard deviation across 5 folds. Brain region combinations were ranked by descending mean validation AUC.

Abbreviations: AUC, area under the receiver operating characteristic curve; SN, substantia nigra; GP, globus pallidus; CN, caudate nucleus; Put, putamen; RN, red nucleus.

Table S5. Performance of logistic regression models using radiomics features from different brain region combinations with 5-fold cross-validation.

Brain Region Combination	No. of Features	AUC	Accuracy	Sensitivity	Specificity
CN+GP	13.2±4.0	0.879±0.045	0.791±0.087	0.780±0.110	0.799±0.132
GP+RN+Put	23.2±2.2	0.851±0.115	0.836±0.095	0.747±0.165	0.907±0.060
SN+GP	12.8±5.9	0.847±0.046	0.810±0.083	0.777±0.120	0.836±0.116
SN+Put	9.6±6.8	0.836±0.078	0.764±0.107	0.762±0.110	0.767±0.153
CN+GP+Put	19.6±3.2	0.833±0.038	0.730±0.050	0.679±0.065	0.771±0.056
CN+Put	16.0±6.2	0.831±0.055	0.769±0.093	0.717±0.192	0.811±0.054
CN+GP+RN+Put	23.0±2.7	0.829±0.111	0.783±0.113	0.800±0.162	0.770±0.100
RN	14.2±6.3	0.827±0.051	0.759±0.053	0.732±0.121	0.781±0.139
GP+RN	16.0±6.3	0.819±0.095	0.722±0.032	0.679±0.088	0.756±0.062
GP	9.8±5.1	0.812±0.045	0.716±0.050	0.748±0.099	0.690±0.057
SN+GP+Put	16.4±7.3	0.812±0.082	0.795±0.106	0.797±0.094	0.793±0.195
RN+Put	18.0±8.2	0.807±0.053	0.761±0.063	0.764±0.107	0.758±0.087
SN+CN+RN	16.4±4.3	0.805±0.067	0.743±0.088	0.745±0.060	0.741±0.159
CN+GP+RN	20.2±3.2	0.805±0.121	0.745±0.070	0.662±0.153	0.810±0.074
CN+RN	19.2±6.8	0.804±0.061	0.692±0.036	0.714±0.108	0.675±0.088
SN+GP+RN+Put	18.4±5.2	0.803±0.087	0.766±0.094	0.712±0.172	0.810±0.092

Brain Region Combination	No. of Features	AUC	Accuracy	Sensitivity	Specificity
SN+CN+GP+Put	19.2±4.1	0.801±0.086	0.743±0.075	0.797±0.094	0.700±0.170
SN+RN	11.2±5.4	0.799±0.090	0.742±0.092	0.779±0.098	0.713±0.156
SN+CN+Put	20.0±3.7	0.798±0.080	0.713±0.063	0.645±0.104	0.768±0.169
GP+Put	16.4±5.0	0.795±0.071	0.746±0.085	0.700±0.192	0.783±0.061
SN+GP+RN	18.4±3.4	0.792±0.134	0.775±0.110	0.782±0.224	0.770±0.103
CN	8.8±5.2	0.788±0.054	0.692±0.024	0.642±0.099	0.731±0.102
Put	11.4±6.8	0.783±0.054	0.716±0.056	0.733±0.160	0.702±0.064
SN+CN	13.8±4.9	0.783±0.096	0.706±0.072	0.679±0.088	0.728±0.141
SN+RN+Put	17.8±8.6	0.781±0.120	0.741±0.148	0.762±0.150	0.725±0.237
SN+CN+GP+RN	21.0±1.0	0.776±0.088	0.758±0.110	0.745±0.060	0.768±0.181
CN+RN+Put	21.6±5.4	0.767±0.042	0.737±0.028	0.694±0.129	0.770±0.058
SN+CN+GP	18.4±3.0	0.754±0.104	0.713±0.094	0.676±0.118	0.742±0.162
SN	8.4±6.0	0.748±0.134	0.675±0.166	0.594±0.205	0.740±0.177
SN+CN+GP+RN+Put	22.4±1.8	0.742±0.032	0.737±0.028	0.695±0.093	0.770±0.079
SN+CN+RN+Put	22.6±3.6	0.738±0.057	0.729±0.074	0.677±0.073	0.770±0.092

Note: Values are presented as mean ± standard deviation across 5 folds. Brain region combinations were ranked by descending mean validation AUC.

Abbreviations: AUC, area under the receiver operating characteristic curve; SN, substantia nigra; GP, globus pallidus; CN, caudate nucleus; Put, putamen; RN, red nucleus.

Table S6. Performance of Support Vector Machine models using radiomics features from different brain region combinations with 5-fold cross-validation.

Brain Region Combination	No. of Features	AUC	Accuracy	Sensitivity	Specificity
GP+RN+Put	23.2±2.2	0.880±0.047	0.827±0.058	0.814±0.137	0.837±0.041
CN+GP	13.2±4.0	0.874±0.044	0.805±0.046	0.712±0.043	0.879±0.072
CN+Put	16.0±6.2	0.871±0.039	0.767±0.066	0.712±0.094	0.810±0.088
CN+GP+RN+Put	23.0±2.7	0.869±0.052	0.820±0.047	0.797±0.043	0.838±0.075
CN+GP+RN	20.2±3.2	0.866±0.018	0.804±0.052	0.779±0.051	0.824±0.078
SN+CN+GP+RN	21.0±1.0	0.862±0.041	0.780±0.101	0.745±0.060	0.809±0.158
CN+GP+Put	19.6±3.2	0.860±0.023	0.790±0.071	0.764±0.089	0.810±0.074
GP+Put	16.4±5.0	0.857±0.050	0.790±0.041	0.714±0.123	0.850±0.059
SN+CN+GP+RN+Put	22.4±1.8	0.855±0.040	0.780±0.078	0.762±0.072	0.795±0.113
SN+CN+GP+Put	19.2±4.1	0.852±0.051	0.750±0.107	0.727±0.078	0.768±0.147
SN+CN+Put	20.0±3.7	0.850±0.054	0.787±0.095	0.761±0.081	0.809±0.108
CN+RN+Put	21.6±5.4	0.847±0.033	0.766±0.037	0.729±0.069	0.796±0.072
SN+CN+RN+Put	22.6±3.6	0.847±0.081	0.748±0.134	0.761±0.081	0.739±0.188
SN+GP+RN+Put	18.4±5.2	0.840±0.040	0.765±0.110	0.797±0.126	0.740±0.157
RN+Put	18.0±8.2	0.839±0.033	0.759±0.038	0.780±0.072	0.742±0.065
SN+RN+Put	17.8±8.6	0.839±0.089	0.763±0.161	0.811±0.119	0.725±0.208

Brain Region Combination	No. of Features	AUC	Accuracy	Sensitivity	Specificity
SN+CN+GP	18.4±3.0	0.833±0.048	0.758±0.104	0.762±0.072	0.754±0.147
SN+GP+RN	18.4±3.4	0.827±0.064	0.771±0.125	0.812±0.140	0.739±0.194
SN+GP+Put	16.4±7.3	0.825±0.085	0.772±0.092	0.780±0.042	0.767±0.191
SN+GP	12.8±5.9	0.822±0.071	0.750±0.085	0.745±0.118	0.753±0.159
CN+RN	19.2±6.8	0.822±0.034	0.743±0.055	0.695±0.125	0.782±0.095
GP+RN	16.0±6.3	0.818±0.082	0.750±0.113	0.709±0.124	0.782±0.116
SN+Put	9.6±6.8	0.816±0.057	0.765±0.093	0.745±0.156	0.781±0.122
SN+CN+RN	16.4±4.3	0.810±0.061	0.719±0.124	0.711±0.055	0.726±0.191
CN	8.8±5.2	0.809±0.027	0.723±0.063	0.611±0.071	0.812±0.144
GP	9.8±5.1	0.796±0.063	0.708±0.076	0.697±0.136	0.717±0.084
SN+CN	13.8±4.9	0.791±0.052	0.721±0.099	0.644±0.069	0.783±0.147
Put	11.4±6.8	0.789±0.111	0.723±0.063	0.717±0.183	0.729±0.073
RN	14.2±6.3	0.778±0.060	0.712±0.090	0.592±0.111	0.808±0.140
SN	8.4±6.0	0.776±0.119	0.659±0.188	0.611±0.279	0.698±0.204
SN+RN	11.2±5.4	0.768±0.071	0.667±0.114	0.679±0.121	0.658±0.220

Note: Values are presented as mean ± standard deviation across 5 folds. Brain region combinations were ranked by descending mean validation AUC.

Abbreviations: AUC, area under the receiver operating characteristic curve; SN, substantia nigra; GP, globus pallidus; CN, caudate nucleus; Put, putamen; RN, red nucleus.

Table S7. Performance of Random Forest models using radiomics features from different brain region combinations with 5-fold cross-validation.

Brain Region Combination	No. of Features	AUC	Accuracy	Sensitivity	Specificity
CN+GP	13.2±4.0	0.891±0.028	0.812±0.034	0.762±0.041	0.852±0.072
CN+GP+RN	20.2±3.2	0.891±0.026	0.781±0.054	0.744±0.124	0.810±0.057
SN+CN+GP+RN	21.0±1.0	0.889±0.023	0.811±0.062	0.747±0.099	0.863±0.089
CN+GP+RN+Put	23.0±2.7	0.878±0.045	0.797±0.081	0.798±0.125	0.796±0.098
CN+RN+Put	21.6±5.4	0.876±0.024	0.760±0.038	0.730±0.064	0.784±0.073
GP+RN+Put	23.2±2.2	0.876±0.051	0.798±0.079	0.833±0.156	0.770±0.034
CN+GP+Put	19.6±3.2	0.875±0.058	0.812±0.034	0.782±0.124	0.837±0.062
CN+Put	16.0±6.2	0.872±0.018	0.797±0.019	0.745±0.060	0.838±0.035
SN+CN+RN+Put	22.6±3.6	0.868±0.047	0.780±0.064	0.795±0.052	0.769±0.085
SN+CN+GP+RN+Put	22.4±1.8	0.865±0.053	0.825±0.074	0.795±0.052	0.850±0.094
SN+CN+GP	18.4±3.0	0.864±0.031	0.773±0.076	0.764±0.107	0.781±0.122
RN+Put	18.0±8.2	0.862±0.083	0.767±0.064	0.782±0.124	0.756±0.078
SN+CN+GP+Put	19.2±4.1	0.859±0.037	0.766±0.082	0.745±0.103	0.782±0.095
CN+RN	19.2±6.8	0.855±0.036	0.775±0.034	0.695±0.110	0.838±0.059
SN+CN+Put	20.0±3.7	0.855±0.037	0.782±0.062	0.764±0.122	0.796±0.072
GP+Put	16.4±5.0	0.854±0.053	0.791±0.064	0.800±0.151	0.784±0.029

Brain Region Combination	No. of Features	AUC	Accuracy	Sensitivity	Specificity
SN+CN+RN	16.4±4.3	0.850±0.060	0.764±0.093	0.711±0.081	0.808±0.140
SN+GP+RN	18.4±3.4	0.847±0.046	0.780±0.090	0.779±0.174	0.781±0.139
SN+CN	13.8±4.9	0.842±0.047	0.759±0.038	0.714±0.090	0.795±0.103
SN+RN+Put	17.8±8.6	0.841±0.043	0.751±0.043	0.695±0.071	0.795±0.103
SN+GP+RN+Put	18.4±5.2	0.840±0.038	0.758±0.068	0.797±0.094	0.728±0.093
GP+RN	16.0±6.3	0.839±0.045	0.774±0.046	0.729±0.036	0.810±0.100
SN+GP+Put	16.4±7.3	0.835±0.039	0.759±0.046	0.729±0.091	0.783±0.102
Put	11.4±6.8	0.834±0.089	0.753±0.063	0.767±0.181	0.743±0.058
SN+GP	12.8±5.9	0.831±0.054	0.728±0.069	0.695±0.110	0.754±0.131
CN	8.8±5.2	0.818±0.026	0.729±0.031	0.661±0.119	0.784±0.087
GP	9.8±5.1	0.804±0.059	0.745±0.095	0.729±0.160	0.757±0.088
RN	14.2±6.3	0.803±0.067	0.705±0.076	0.694±0.099	0.714±0.097
SN+Put	9.6±6.8	0.797±0.073	0.743±0.055	0.695±0.161	0.782±0.106
SN+RN	11.2±5.4	0.783±0.088	0.727±0.094	0.729±0.069	0.727±0.180
SN	8.4±6.0	0.749±0.094	0.646±0.132	0.580±0.239	0.699±0.187

Note: Values are presented as mean ± standard deviation across 5 folds. Brain region combinations were ranked by descending mean validation AUC.

Abbreviations: AUC, area under the receiver operating characteristic curve; SN, substantia nigra; GP, globus pallidus; CN, caudate nucleus; Put, putamen; RN, red nucleus.

Table S8. Performance of XGBoost models using radiomics features from different brain region combinations with 5-fold cross-validation.

Brain Region Combination	No. of Features	AUC	Accuracy	Sensitivity	Specificity
GP+RN+Put	23.2±2.2	0.921±0.040	0.805±0.046	0.798±0.093	0.810±0.032
CN+GP+RN+Put	23.0±2.7	0.919±0.034	0.812±0.057	0.782±0.110	0.837±0.062
CN+GP+Put	19.6±3.2	0.893±0.041	0.805±0.027	0.762±0.041	0.839±0.058
SN+CN+GP+RN	21.0±1.0	0.891±0.031	0.849±0.041	0.814±0.069	0.877±0.090
SN+CN+RN+Put	22.6±3.6	0.890±0.031	0.826±0.048	0.797±0.043	0.850±0.094
GP+Put	16.4±5.0	0.886±0.080	0.776±0.096	0.767±0.181	0.784±0.073
CN+GP+RN	20.2±3.2	0.885±0.018	0.813±0.072	0.764±0.122	0.852±0.072
SN+CN+GP+RN+Put	22.4±1.8	0.885±0.032	0.819±0.038	0.795±0.052	0.837±0.062
CN+RN+Put	21.6±5.4	0.873±0.027	0.782±0.050	0.747±0.080	0.810±0.113
CN+GP	13.2±4.0	0.872±0.032	0.775±0.057	0.711±0.055	0.826±0.100
GP+RN	16.0±6.3	0.869±0.088	0.814±0.089	0.817±0.124	0.811±0.086
SN+GP+RN	18.4±3.4	0.864±0.044	0.780±0.058	0.795±0.079	0.769±0.085
SN+GP+RN+Put	18.4±5.2	0.863±0.036	0.752±0.041	0.780±0.093	0.730±0.048
CN+RN	19.2±6.8	0.859±0.028	0.774±0.059	0.730±0.105	0.810±0.079
RN+Put	18.0±8.2	0.858±0.054	0.753±0.027	0.782±0.071	0.730±0.009
CN+Put	16.0±6.2	0.857±0.023	0.781±0.070	0.779±0.128	0.783±0.061

Brain Region Combination	No. of Features	AUC	Accuracy	Sensitivity	Specificity
SN+CN+GP+Put	19.2±4.1	0.857±0.034	0.804±0.031	0.780±0.042	0.824±0.040
RN	14.2±6.3	0.854±0.061	0.796±0.056	0.764±0.089	0.822±0.100
SN+CN+GP	18.4±3.0	0.852±0.033	0.774±0.038	0.730±0.064	0.810±0.063
SN+CN+RN	16.4±4.3	0.844±0.062	0.758±0.077	0.727±0.078	0.782±0.106
SN+CN+Put	20.0±3.7	0.843±0.042	0.759±0.038	0.712±0.094	0.796±0.109
SN+RN+Put	17.8±8.6	0.833±0.047	0.743±0.066	0.695±0.071	0.782±0.095
SN+GP+Put	16.4±7.3	0.829±0.046	0.789±0.058	0.764±0.122	0.810±0.063
SN+GP	12.8±5.9	0.825±0.044	0.788±0.075	0.797±0.172	0.781±0.131
SN+CN	13.8±4.9	0.818±0.054	0.683±0.102	0.701±0.058	0.726±0.108
CN	8.8±5.2	0.807±0.047	0.743±0.060	0.759±0.138	0.730±0.048
GP	9.8±5.1	0.806±0.061	0.692±0.058	0.662±0.114	0.716±0.087
Put	11.4±6.8	0.804±0.111	0.709±0.102	0.771±0.158	0.729±0.055
SN+Put	9.6±6.8	0.803±0.071	0.692±0.097	0.682±0.122	0.762±0.045
SN+RN	11.2±5.4	0.784±0.087	0.721±0.083	0.645±0.146	0.782±0.126
SN	8.4±6.0	0.733±0.142	0.639±0.115	0.547±0.243	0.713±0.163

Note: Values are presented as mean ± standard deviation across 5 folds. Brain region combinations were ranked by descending mean validation AUC.

Abbreviations: AUC, area under the receiver operating characteristic curve; SN, substantia nigra; GP, globus pallidus; CN, caudate nucleus; Put, putamen; RN, red nucleus.
